# Transparent PC/PMMA Blends with Enhanced Mechanical Properties via Reactive Compounding of Functionalized Polymers

**DOI:** 10.3390/polym14010073

**Published:** 2021-12-25

**Authors:** Tobias Bubmann, Andreas Seidel, Holger Ruckdäschel, Volker Altstädt

**Affiliations:** 1 Department of Polymer Engineering, University of Bayreuth, Universitätsstraße 30, 95447 Bayreuth, Germany; tobias.bubmann@uni-bayreuth.de (T.B.); ruckdaeschel@uni-bayreuth.de (H.R.); 2 Covestro Deutschland AG, Business Entity Engineering Plastics, Research & Development, Development Blends, 51365 Leverkusen, Germany; andreas.seidel@covestro.com; 3 Bavarian Polymer Institute and Bayreuth Institute of Macromolecular Research, University of Bayreuth, Universitätsstraße 30, 95447 Bayreuth, Germany

**Keywords:** reactive extrusion, PC, PMMA, transparency, blend, compatibilization, morphology, structure–property relationships, mechanical properties, PC/PMMA copolymer

## Abstract

Reactive compounding of terminally phenolic OH-functionalized polycarbonate (PC) with epoxy-functionalized polymethylmethacrylate (PMMA) prepared by copolymerization with glycidyl methacrylate was investigated. It was spectroscopically demonstrated that a PC/PMMA copolymer was formed during the melt reaction of the functional groups. Zirconium acetylacetonate could catalytically accelerate this reaction. Correlations of the phenomenological (optical and mechanical) properties with the molecular level and mesoscopic (morphological) structure were discussed. By the investigated reactive compounding process, transparent PC/PMMA blends with two-phase morphologies were obtained in a continuous twin-screw extruder, which, for the first time, combined the high transmission of visible light with excellent mechanical performance (e.g., synergistically improved tensile and flexural strength and high scratch resistance). The transparency strongly depended on (a) the degree of functionalization in both PC and PMMA, (b) the presence of the catalyst, and (c) the residence time of the compounding process. The in-situ-formed PC/PMMA copolymer influenced the observed macroscopic properties by (a) a decrease in the interphase tension, leading to improved and stabilized phase dispersion, (b) the formation of a continuous gradient of the polymer composition and thus of the optical refractive indices in a diffuse mesoscopic interphase layer separating the PC and PMMA phases, and (c) an increase in the phase adhesion between PC and PMMA due to mechanical polymer chain entanglement in this interphase.

## 1. Introduction

Polymer blending is a simple, versatile, and economical tool for developing new polymer materials with tailored properties. By combining the advantages of different polymers or even, in favorable cases, by exploiting the property synergies of the blend partners, polymer blends can fulfill the complex requirements of many industrial applications [1,2,3]. For example, the automotive industry plays a key user role in the polymer blend market [4]. The main advantages of tailor-made polymer blends, compared to developing new polymers, are the short time-to-market and typically no investment needed for scale-up, so that the barriers to successful market introduction of new products are essentially lower.

In particular, blends based on polycarbonate (PC) are of great commercial interest. In addition to the improvement of already industrially established PC blends such as PC/acrylonitrile–butadiene–styrene (ABS), PC/styrene–acrylonitrile (SAN), or PC/polybutylene terephthalate (PBT), the development of novel PC blends with polymeric blend partners not yet used industrially still has enormous scientific, technical, and economic potential. For example, blending PC with polymethyl methacrylate (PMMA) is believed to have the potential to overcome some of the technical shortcomings of PC, such as scratch sensitivity, chemical resistance, or birefringence, while retaining its valued unique advantages such as good heat and impact resistance. The main challenge with this blend system is the loss of transparency, which is the main selling point of the pure polymers. The opacity that is usually observed with blends compounded from commercial transparent PC and PMMA grades is due to the immiscibility of these polymers at most composition ratios [5]. 

Various process strategies (i.e., solution casting or melt mixing) have been reported in the literature to produce transparent or translucent PC/PMMA blends. Since the preparation of polymer blends by solution casting [6,7,8,9,10,11,12,13,14,15] is not an industrially relevant process, the present study is limited to the compatibilization of melt-mixed PC/PMMA blends.

In general, two different concepts of phase compatibilization can be distinguished, namely physical and chemical approaches. In chemical (or reactive) compatibilization processes, chemical reactions take place at the interphases of the blend partners during melt mixing, leading to the in-situ formation of phase compatibilizers. In contrast, no reaction takes place in physical compatibilization. There are only a few reports in the literature on physical compatibilization leading to transparent PC/PMMA blends by melt mixing. Orlando et al. [16], for example, report a transparent PC/PMMA blend (disclosed to be commercialized under the trade name Iupilon MB6001UR) that exhibits technical properties such as flexural strength and scratch resistance that are intermediate to those of the pure blend partners. The transparency in this case is claimed to result from the enhanced miscibility of the two polymers, which is achieved by using a PMMA copolymer instead of pure PMMA. The chemical nature of the copolymer is not explained in detail. To the best of our knowledge, the method of using pre-synthesized copolymers as compatibilizers in melt-mixed PC/PMMA blends has not been investigated. The third established method of physical compatibilization is the addition of nanoparticles that accumulate at the polymer/polymer interphases, reducing the interfacial tension. There are three known publications reporting that transparent PC/PMMA blends have been prepared based on this approach [17,18,19]. Dhibar et al. [17] investigated the transparency of PC blends with in-situ suspension-polymerized and exfoliated PMMA/clay nanocomposites. The mechanical properties of the obtained blends were not reported. Singh et al. [18] investigated the effect of adding 3 wt% Closite15A to PC/PMMA blends of different compositions on their transparency and mechanical properties and found an increasing improvement in polymer miscibility and consequently material transparency at higher PMMA content in the blend. Xi et al. [19] used Aerosil A2200 to modify the interfacial surface tension in a PC/PMMA (80/20) blend. The size of the dispersed PMMA domains could be significantly reduced with 3 wt% of the nanoparticles. However, the transparency of the blend system changed only slightly compared to the neat blend.

Regarding the chemical (reactive) compatibilization of PC/PMMA blends, two different approaches can be distinguished. The first approach uses commercially available unmodified PC and PMMA polymers as blend partners and relies on the formation of copolymers by transesterification between carbonate groups in the polymer backbone of the PC with methacrylate ester groups in the PMMA. This transesterification approach has been intensively studied and scientifically published [20,21,22,23,24], as well as disclosed in patent applications [25,26,27]. The serious drawback of this reactive compatibilization strategy is that the reaction leading to the formation of the desired copolymer results in a significant molecular weight degradation in the PC. Therefore, the mechanical performance of the obtained blends is poor, rendering them useless for any industrial applications [21]. The second approach to reactive compatibilization is to reactively modify both blend partners (in this case, PC and PMMA) with functional groups. These can then be covalently connected in a melt reaction to form in-situ PC/PMMA copolymers. This second approach has been described for other polymer combinations, but, to the best of our knowledge, has not yet been pursued for the reactive compatibilization of PC/PMMA blends. Presumably, the reason for this is that commercial PC grades typically do not contain appreciable amounts of reactive phenolic hydroxyl (pOH) end groups, contrary to the misconception often asserted in the scientific literature [28,29]. Industrial manufacturers of PC generally take care to minimize the content of pOH end groups. The presence of such groups adversely affects the material properties of the polycarbonate. Therefore, the use of commercially available PC grades generally does not result in any significant yields in the melt coupling reaction with reactive functionalized blend partners unless pOH is formed in-situ in the reactive compounding, i.e., by hydrolysis of the PC in the presence of water and catalysts. However, such hydrolysis leads to an undesirable reduction in the molecular weight of PC and thus to a deterioration in the mechanical properties of the material. 

With the present work, we intend to close the described scientific knowledge gap by investigating the reactive compounding of tailor-made pOH-functionalized PC with PMMA random copolymers containing small amounts of glycidyl methacrylate (GMA) co-monomer units. The working hypothesis was that, during melt compounding, the epoxy (EP) groups of the GMA units react—hopefully fast enough—with the pOH end groups of the PC. During this reaction, in-situ PC/PMMA copolymers are formed in such a way that (several) PC blocks are grafted onto a PMMA backbone. These copolymers would then act as phase compatibilizers in the PC/PMMA blend. The reaction seems very plausible as it has already been described in several publications for other polymer systems [30,31,32]. 

The main questions that we aim to answer with this study are (a) whether the reactive extrusion of pOH-functionalized PC with GMA-modified PMMA is a suitable strategy for the preparation of transparent PC/PMMA blends with improved mechanical performance using industrially established compounding equipment; (b) whether the reaction kinetics of the two-phase melt reaction is fast enough, even at the short residence times (RT) typical of industrially attractive continuous twin-screw compounding processes, to achieve sufficiently high yields of PC/PMMA copolymers; (c) whether catalysts can accelerate the polymer coupling reaction and thus improve copolymer yields and material performance; and (d) how the degree of functionalization of PC and PMMA affects the polymer blend performance. By correlating the macroscopic engineering properties with the mesoscopic (morphological) and microscopic (molecular) structure of the blends, we also aim to develop a better mechanistic understanding of the structure–property relationships, i.e., of the structural requirements for transparency and improved mechanical performance in PC/PMMA blends.

## 2. Materials and Experimental

### 2.1. Materials 

All PC materials were obtained from Covestro Deutschland AG (Leverkusen, Germany). Both non-modified PC grades are commercial products. PC* represents Makrolon^®^ 2408; PC** is a physical mixture of 30 wt% Makrolon^®^ 2408 and 70 wt% Makrolon^®^ OD2015. The mass ratio of the two PC grades was chosen in such a way as to obtain the same average molecular weight (M_w_) as for the functionalized m-PC**. The pOH-functionalized PCs (i.e., m-PC* and m-PC**) were prepared by tailor-made synthesis via melt polymerization of diphenyl carbonate in a stoichiometric excess of bisphenol A. The prefix “m” in our sample nomenclature denotes reactively “modified” samples. Table 1 summarizes, for all PC materials, the M_w_ and M_n_ determined by gel permeation chromatography (GPC), as well as the contents of terminal pOH groups determined by ^1^H NMR spectroscopy. The molecular weight values in Table 1 are reported with polystyrene calibration (please note: M_w_ values measured against BPA-PC calibration are smaller by a factor of roughly 1.7 and M_n_ values by a factor of 1.7 for commercial and 2.1 for m-PC, respectively, compared to the PS calibrated values). The commercial (non-modified) PCs contain negligible amounts of pOH groups (<0.01 wt% corresponding to <5 mol% of the total polymer end groups). The pOH contents of the modified PC grades m-PC* and m-PC**, on the other hand, in both cases, correspond approximately to a level of 70 ± 5 mol% of the polymer end groups. Assuming statistical distribution of end groups, this means that, in the modified m-PCs, roughly equal portions of the polymer chains will contain one and two pOH end groups, respectively, and only less than 10% of the PC molecules will not contain any pOH end group. 

The unmodified PMMA (Plexiglas^®^ POQ62) was sourced from Evonik Industries AG (Essen, Germany). The modified m-PMMAs are random PMMA-co-GMA copolymers of methyl methacrylate (MMA) with glycidyl methacrylate (GMA) and were kindly tailor-synthesized and provided by Fine-Blend Polymer Co., Ltd. (Shanghai, China). The two materials, m-PMMA1 and m-PMMA8, were distinguished by their GMA contents of 1 wt% and 8 wt%, respectively, and were both designed to match the molecular weight distribution of the unmodified PMMA. The molecular weights of the PMMAs (determined by GPC and reported against PS calibration) as well as their EP contents (determined by the titration method according to ASTM D1652 in dichloromethane solution) are also summarized in Table 1. The GMA contents of m-PMMA1 and m-PMMA8 corresponded to averages of 2 and 18 EP functionalities per polymer chain in the respective materials. 

The material SAG8 used in the kinetic model experiments represented commercial GMA-modified styrene–acrylonitrile (SAN) copolymer Fineblend^®^ SAG-008, sourced from Fine-Blend Polymer Co., LTD (Shanghai, China). The polymer contained 8 wt% of GMA, which was randomly copolymerized with the styrene and acrylonitrile (mass ratio of styrene and acrylonitrile at approximately 75:25). Molecular weights and EP contents of SAG8 are summarized in Table 1. The EP content of SAG8 was the same as for m-PMMA8. Due to the higher molecular weight of the SAG8 compared to m-PMMA8, this, however, corresponded to a larger average EP functionality per polymer chain of 32 in the SAG8.

For the fundamental investigation of the melt reaction kinetics of EP-containing polymers (see Section 2.2.1), 4-cumylphenol (CP), 1,1-diphenylethanol, diphenylmethanol, 1-naphthalenemethanol, and diphenyl carbonate were used as model compounds to study reactivity with pOH, tertiary (tert.), secondary (sec.), and primary (prim.) aliphatic (a)OH and carbonate functional groups, respectively. The sources of the chemicals as well as their boiling points T_B_ are compiled in Table 2. 

The zirconium (IV) acetylacetonate (ZrAcac) used in this investigation as a catalyst was the commercial product Catana^TM^ CAA 2950 (Sachem, Inc., Austin, TX, USA). Catalyst p-toluene sulfonic acid monohydrate (p-TSA) was sourced from Merck KGaA (Darmstadt, Germany). 

### 2.2. Experimental

#### 2.2.1. Model Study of Melt Reaction Kinetics

**Figure 1:**
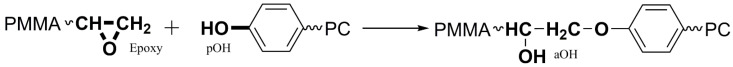
Scheme of the PC/PMMA copolymer forming melt reaction of the EP groups in GMA-functionalized PMMA with pOH end groups in reactively modified PC. A secondary aliphatic OH (sec. aOH) group is formed during this reaction.

Systematic studies of the kinetics of the reaction of EP groups containing compounds with reactants of different functionalities reported in the literature [30,31,33] have been limited to reactions in solution at rather low temperatures (<100 °C) and long reaction times (hours). It was unclear to what extent the conclusions of these studies could be reliably transferred to the physical conditions of the melt compounding that are relevant to our work (i.e., melt mixtures of polymers at temperatures of around 260 °C under relatively low shear rates on the order of 100 s^−1^ and with short RT in the range of a few minutes). Additional aspects contributing to the complexity of the reaction kinetics in melt mixtures of reactively modified immiscible polymers are the typically very low concentrations of the reactive groups and the fact that the reaction can only occur at the interphases of the polymer domains in the two-phase melt mixtures. This means that the experimentally observed reaction kinetics might be strongly affected, if not even essentially determined, by (a) the quality of phase dispersion in the melt mixture, i.e., the extent of interphase formation (and thus dependent on process parameters such as melt temperature, melt viscosity, shear rates, presence of dispersion aids, etc.); (b) by the diffusion coefficients of the polymers in their respective phase domains and thus by the velocity of interphase renewal and, finally, (c) by the three-dimensional conformational structure (inter- and intramolecular entanglement) adopted by the two polymers forming the blend in the polymer–polymer interphase (the latter influencing the sterical accessibility of the functional groups for reaction). To gain better insight into the individual molecular-level contributions to measurable reactivity, we started with a kinetic model study. Here, we investigated the reactivity of relevant EP-group-containing polymers under ideal melt conditions, i.e., in homogeneous (single phase) melt mixtures with low-molecular-weight chemical compounds of different functionality. By suppressing the effects of phase dispersion and diffusion processes on the reaction kinetics, we aimed to allow the evaluation of the “ideal” (i.e., maximum achievable) reaction velocity and reaction yield under melt compounding conditions. Our intention was to determine which reaction functionality would be best suited for the intended purpose before eventually engaging in the analogous, more complex reactions in two-phase melt mixtures of immiscible polymers. In addition, we aimed to use these model studies to assess whether a catalyst would be required to achieve tangible reaction yields and to develop a basic understanding of potentially competing (undesirable) side reactions of the EP-containing polymer with aliphatic OH groups through reliable analytical monitoring. The aliphatic OH groups would eventually be formed in the targeted reaction of pOH-functionalized PC with EP-functionalized PMMA/GMA copolymers (see Figure 1). 

The compounds used as reactants in the model studies (see Table 2) were selected to meet the following three requirements: (a) structural similarity to PC, i.e., similar polarity to the eventually used polymer reaction partner, (b) sufficient thermal stability and high boiling point T_B_ to allow investigation of melt reactions at compounding temperatures (260 °C and above) without significant volatilization and/or degradation of the reactant compound, and (c) monofunctionality, i.e., the reactant should contain only a single reactive group. Monofunctional reactants were considered necessary to prevent possible crosslinking. The latter would complicate the analytical monitoring and quantification of the yield of the target reaction, since crosslinking usually affects the solubility of a polymer, which is an important prerequisite for suitable analytical techniques. However, to allow the analytical monitoring of reaction kinetics, i.e., target conversion vs. time, a relatively high content of polymer functionality is required as the detection limits of suitable analytical techniques are limited. It was therefore necessary to use polymers with a relatively high EP content and thus a high number of reactive groups.

Most of the kinetic model studies were performed with commercially available SAG8, which contains aromatic styrene as the dominant monomer constituent. All low-molecular-weight reactants were aromatic compounds, ensuring complete miscibility with SAG8 in the melt mixture. However, the aromatic reactants were also found to be miscible with the m-PMMA grades at the ratios used in the kinetic study. We hoped that the aromatic model reactants would be the most suitable simulants for the aromatic (BPA-based) PC that we would use for the eventually targeted polymer–polymer melt reactions later on.

Prior to compounding, the polymers and reactant model compounds were pre-dried overnight in vacuum at 60 °C and at room temperature, respectively. Kinetic studies were performed in a discontinuously running micro-compounder, MC15 from Xplore Instruments BV (Sittard, Netherlands), using RTs in the range of 5 to 60 min and a melt temperature of 260 °C (rotational screw speed at 100 rpm). In preparation for compounding, the polymers were ground into powders and premixed with the reactant model compounds in amounts corresponding to the equimolar ratios of EP groups of the GMA-modified polymer to functional groups of the model compounds. If catalysts were used, they were added to this powder premix in an amount of 0.05 wt%.

Reaction conversions were monitored semi-quantitatively by measuring the force over time in the micro-compounder and quantitatively after several reaction times by determining the changes in EP content in the reaction mixture by titration according to ASTM D1652 in dichloromethane solution with potentiometric detection. The principle of this titration method is based on the reaction of nascent hydrogen bromide, formed by the action of perchloric acid on tetra-ethyl ammonium bromide, with the EP groups present in the investigated sample. Moreover, in the case of CP as a model compound, the reduction of the content of this reactant in the reaction mixture was studied. For quantitative analysis of residual (i.e., unreacted) EP groups and CP, the reaction was quenched by rapidly cooling the reaction mixture to room temperature. For CP quantification the cooled reaction mixture was extracted using tetrahydrofuran (THF) and then the solved CP precipitated with a polar solvent. The resulting suspension was purified and afterwards chromatographed applying high-pressure liquid chromatography (HPLC) with UV detection. Quantification was performed against an external standard on a reversed-phase column. Both the titration of the EP content and the determination of the residual content of CP by HPLC were carried out by Currenta GmbH & Co. OHG (Leverkusen, Germany).

#### 2.2.2. Compounding and Reactive Blending of PC and PMMA

**Figure 2:**
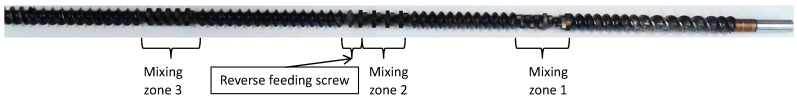
Screw profile of the used screws in the continuous parallel twin-screw extruder showing the three mixing zones.

PC/PMMA blends with mass ratios of PC and PMMA of (80/20) and (50/50) were prepared by melt extrusion using twin-screw extruders of different sizes. The discontinuously running micro-compounder, MC15 from Xplore Instruments BV (Sittard, Netherlands), was used with RTs ranging from 1 to 15 min. Compounding conditions were set to a melt temperature of 260 °C and a rotation speed of 100 rpm. For continuous reactive extrusion, a Process 11 parallel twin-screw extruder from Thermofisher Scientific (Waltham, MA, USA) was used with the same melt temperature of 260 °C but a much lower RT of approximately 1.5 min. The screw profile of the parallel twin-screw extruder is shown in Figure 2. Prior to compounding in both machines, blend components were grounded to fine powder using the Retsch ultra centrifugal mill ZM 100 (Retsch GmbH, Haan, Germany) to prepare a powder premix. The catalyst content was fixed at 0.05 wt% in all experiments where a catalyst was used.

#### 2.2.3. Preparation of Test Specimens

The test specimens were made either by hot pressing or by injection molding. Hot pressing was used to produce 1 mm thick round plates with a diameter of 25 mm and 30 mm × 6 mm × 1 mm test bars from the blends prepared in the MC15 micro-compounder. For hot pressing, the compounds were first plasticized under non-pressurized conditions at a temperature of 260 °C for 2 min, then compressed at the same temperature with a pressure of 60 bar for another 2 min, and finally transferred to a cold press to cool down within 30 s at 20 bar. Injection molding was used to produce 80 mm × 80 mm × 1 mm plates, 80 mm × 10 mm × 4 mm test bars, and dog-bone-type tensile bars with dimensions of 120 mm × 8 mm × 3 mm from compounds produced on the P11 continuous twin-screw extruder. Injection molding was performed on an Arburg 470 H 1000-170 (Arburg GmbH & Co. KG, Loßburg, Germany) injection molding machine with a melt temperature of 270 °C, a mold temperature of 60 °C, and an injection pressure of 2000 bar. RT of the material in the injection molding machine was around 3 min.

#### 2.2.4. Assessment of Optical Properties

Transparency and color were qualitatively assessed by visual inspection and transmission in addition quantitatively determined according to DIN EN ISO 13468-1 using 1 mm thick specimens.

#### 2.2.5. Transmission Electron Microscopy (TEM)

The morphological characterization was performed via bright-field TEM using a Zeiss EM922 OMEGA (Oberkochen, Germany) at an acceleration voltage of 200 kV. Ultrathin sections (~50 nm) were prepared from the compounded granulates or molded test specimens using an ultramicrotome Leica EM UC7 (Wetzlar, Germany). The ultrathin sections were stained with ruthenium tetroxide (RuO_4_) for 15 min to enhance the contrast of the constituent polymers. After such treatment, the PC appears as a darker phase in the TEM micrographs.

#### 2.2.6. Dynamic Mechanical Analysis (DMA)

The DMA was performed using the dual-cantilever method on the Mettler Toledo DMA/SDTA 821e (Columbus, Ohio, USA) with the major intention to investigate changes in the glass transition temperatures (T_g_) of the blend components, which are indicative of efficient phase compatibilization of the blended polymers. For this purpose, hot-melt-pressed specimens with dimensions of 30 mm × 6 mm × 1 mm were used. The measurements were performed under tension at a frequency of 1 Hz. The strain was adjusted to ensure that the complete measurement was performed in the linear-elastic range. Measurements were performed with a constant heating rate of 2 K/min in the temperature range of 25 °C to 160 °C.

#### 2.2.7. Fourier Transform Infrared Spectroscopy (FTIR)

FTIR was used to prove and semi-quantitatively estimate yields of formation of PC/PMMA copolymer upon compounding of the PC/PMMA blend compositions with reactively functionalized polymers. For this purpose, approximately 3 g of the compounded PC/PMMA blends was extracted in 100 mL of acetone under stirring for 24 h at room temperature, followed by filtration with a Büchner funnel to separate the acetone-soluble PMMA part of the blend from the acetone-insoluble PC part. Afterwards, the materials were dried to remove the residual acetone. The acetone-soluble and -insoluble fractions of the products were separately analyzed by FTIR with an FTIR spectrometer, the Nexus 470 from Nicolet (Thermofisher Scientific) (Waltham, MA, USA), in attenuated total reflection (ATR) mode. FTIR spectra were recorded in the range of 400–4000 cm^−1^ with a resolution of 1 cm^−1^. FTIR bands at 1725 cm^−1^ and 1775 cm^−1^ were related to C = O stretching vibrations, which are characteristic of PMMA and PC, respectively. In purely physical mixtures of PC and PMMA, i.e., as produced by melt compounding of non-reactive blend partners in absence of any catalyst, the complete separation of the two blend components by this extraction procedure had been proven by us [21]. Thus, any presence of PMMA in the acetone-insoluble part as indicated by an absorption band at 1725 cm^−1^ in its FTIR spectrum is evidence for a chemical reaction that results in the changed solubility of the PMMA in acetone and thus is strongly indicative of PC/PMMA graft copolymer formation.

#### 2.2.8. Nuclear Magnetic Resonance Spectroscopy (NMR)

^1^H NMR was used to quantify the content of PC/PMMA graft copolymer in the reactively compatibilized PC/PMMA blends. For this purpose, ^1^H NMR spectra of the acetone-insoluble parts of different blends were recorded. First, 30 mg of the acetone-insoluble part of the investigated compound was dissolved in approximately 0.8 mL deuterated chloroform. All investigated samples were found to be completely soluble in chloroform. This proved that the acetone-insoluble parts of the blends did not contain any significantly crosslinked PMMA, which would not only be insoluble in acetone, but in chloroform as well. Thus, any presence of PMMA in the acetone-insoluble part of the reactively compounded blends could be regarded as scientific proof of the formation of PC/PMMA graft copolymer. ^1^H NMR spectra were recorded with a Bruker Avance spectrometer (Billerica, MA, USA) with 300 MHz at room temperature. ^1^H NMR signals at 3.6 ppm (singlet related to three methyl ester protons of MMA in PMMA) and 1.7 ppm (singlet related to six methyl protons of bisphenol A units in PC) and in the range of 7.1–7.3 ppm (multiplet attributed to eight aromatic protons of bisphenol A units in PC) were integrated and used for the quantification of PMMA content in the acetone-insoluble part of the compound. 

#### 2.2.9. Mechanical Properties 

Tensile testing was performed using the dog-bone injection-molded test specimens at room temperature in accordance with ISO 527, with a test speed of 5 mm/min (and 1 mm/min used for Young’s modulus measurement), using a tensile testing machine Z020 from Zwick (Ulm, Germany). 

The flexural strength and bending modulus were measured by means of 3-point bending tests according to ISO 178 using the injection-molded test bars of dimensions 80 mm × 10 mm × 4 mm. For this purpose, the ZMART.PRO Z1485 testing machine from Zwick/Roel (Ulm, Germany) was used with a 10 kN load cell. The center deflection was measured by means of the crosshead travel. The test speed and the speed for measuring the bending modulus were 2 mm/min. 

For assessment of scratch resistance, the pencil hardness was measured using a scratch hardness tester, the TriForcePencil 293 from Erichsen (Hemer, Germany). The measurement was performed according to the Erichsen test specifications using injection-molded plaques of dimensions 80 mm × 80 mm × 1 mm as test specimens. The pencils were clamped in a fixture at an angle of 45° to the test surface and pushed over the specimen surface with a force of 5 N. The hardness of the first pencil (starting with the pencil of highest hardness), whose tip no longer left a perceptible scratch on the test specimen, was considered as the scratch resistance characterizing parameter (the “pencil hardness” of the material).

## 3. Results and Discussion

### 3.1. Model Study of Reaction Kinetics of EP Conversion in Polymer Melts

Table 3 summarizes the complete experimental results of the model study experiments targeting the assessment of the reaction kinetics of EP conversion in single-phase polymer melt mixtures. 

**Figure 3:**
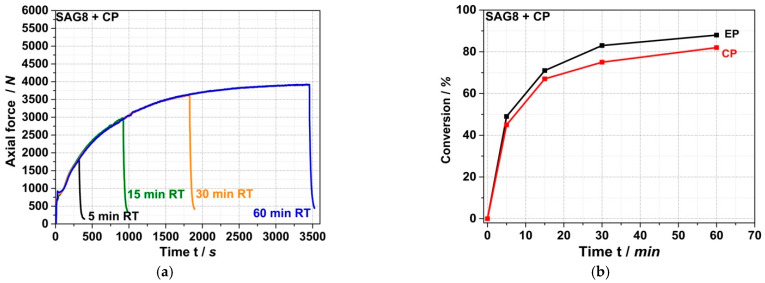
Force vs. time curves (**a**) measured during compounding of a reactive melt mixture of SAG8 and CP with a stochiometric ratio of the EP groups in the SAG8 and the CP. Compounding was performed at a melt temperature of 260 °C in the MC15 micro-compounder. Different colored curves in (**a**) show results of four experimental repetitions, which were stopped after different reaction times to allow determination of the conversion of both EP groups and residual (unreacted) CP in the reaction mixture. The conversion yields of EP and CP are plotted vs. reaction time in (**b**).

Figure 3a shows the force vs. time curves measured during compounding at 260 °C for up to 60 min by the force progression of the MC15 micro-compounder in a reactive melt mixture of SAG8 with CP with a stoichiometric ratio of EP and pOH groups. 

A well-reproducible exponential increase in force over time is observed, converging asymptotically to a constant value at a reaction time of around 60 min. This behavior indicates a relatively slow chemical reaction that takes place during compounding, leading to an increase in the viscosity of the melt. This reaction approaches completion or chemical equilibrium within approximately one hour. Figure 3b shows the conversions of EP groups and CP measured in the reaction mixtures after quenching the reaction by rapid cooling to room temperature at different reaction times. A typical kinetic reaction curve can be seen, with very similar conversions of EP and CP, both converging exponentially to 80–90% after around 60 min. The shape of the conversion vs. time curve shown in Figure 3b is similar to the shape of the force versus time curve shown in Figure 3a. Thus, the results presented in Figure 3a,b provide evidence that a reaction occurs involving both the EP groups and the CP that causes an increase in the viscosity of the melt. Our interpretation of this result is that the CP is grafted onto the SAG8 backbone via the nucleophilic addition of the pOH group of the CP to the EP rings in the SAG8 polymer.

**Figure 4:**
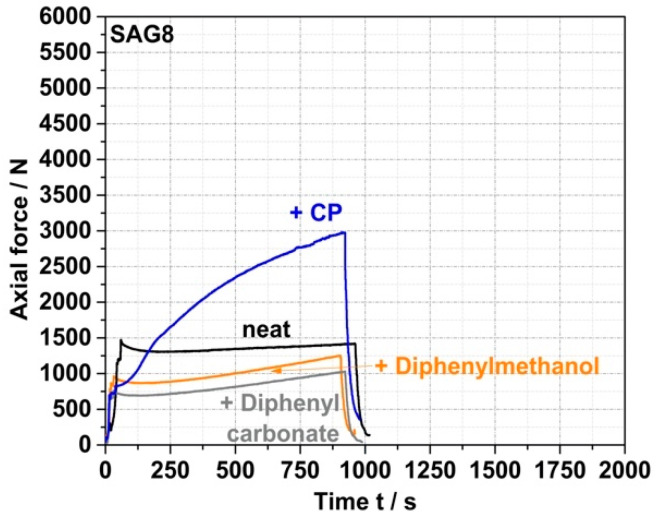
Force vs. time curves measured during compounding of melt mixtures of SAG8 and model reactants of different reactive functionality in the absence of any catalyst. SAG8 and the model reactants were melt-mixed in ratios equivalent to a stoichiometric ratio of the EP groups in the SAG8 and the monofunctional model reactants.

On the other hand, the data in Table 3 show that the EP conversion yields observed under the same process conditions with model compounds containing aOH rather than pOH groups are much lower (<10% for a reaction time of 15 min for both prim., sec., and tert. aOH). Moreover, the force vs. time curves recorded for the reaction mixtures based on these model compounds do not show any increase in force similar to the CP case (see Figure 4 for the specific example of the reaction of SAG8 with diphenylmethanol containing sec. aOH groups), which would indicate a melt reaction of the aOH-containing model compounds with the SAG8. Undesirable side reactions that could have been expected between the EP groups of the GMA-modified polymers and the sec. aOH groups formed in a previous reaction according to Figure 1 can thus be neglected in the absence of a catalyst.

Side reactions of the EP groups with carbonate functional groups in the PC backbone can also be excluded based on the negligible EP conversion (Table 3) and force increase determined in the model experiment with diphenyl carbonate as a reactant (Figure 4). The fact that no EP conversion is observed in the pure SAG8 under the same high-temperature melt conditions (Table 3) proves that a reaction between two EP groups or a reaction between an EP group and a nitrile group in SAG8, which would lead to undesired crosslinking, can also be excluded as side reactions. 

Since the low-molecular-weight reactants were used in equimolar amounts of the EP groups in the model experiments, and since both EP groups and CP were apparently mostly converted within 60 min, most of the CP molecules must have been chemically bonded to the SAG8 backbone in the melt reaction. Low-molecular-weight compounds homogeneously dissolved in a polymer typically cause the polymer to soften and thus reduce its melt viscosity. On the other hand, a chemical reaction such as the grafting of the CP onto the SAG8 polymer will increase its molecular weight and thus the melt viscosity (note that, with an average of 32 GMA units per polymer chain in SAG8, complete conversion of the EP groups with CP will significantly increase the molecular weight of SAG8 by approximately 6800 g/mol). The increase in force over time observed in the micro-compounder in the experiment with CP can thus be easily understood as a direct consequence of the grafting reaction. On the other hand, the lower force observed in the melt mixtures of SAG8 with the non-reactive model compounds compared to the pure SAG8 is a consequence of the softening effect. 

**Figure 5:**
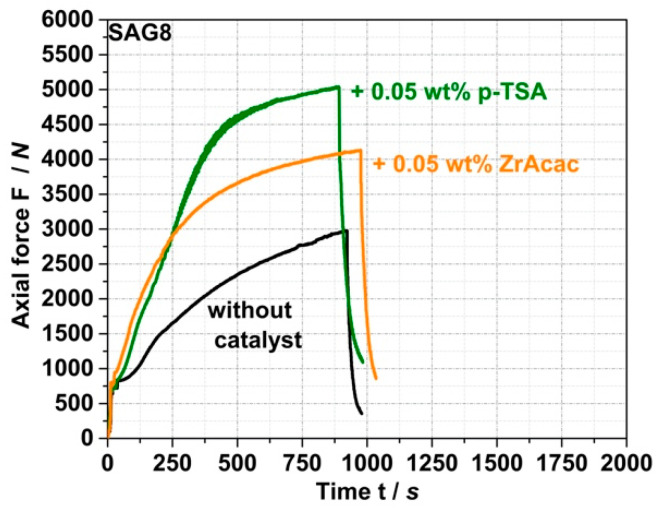
Force vs. time curves measured during compounding of a melt mixture of SAG8 and CP with a stoichiometric ratio of the EP groups in the SAG8 and the CP both in absence of a catalyst as well as in presence of 0.05 wt% of ZrAcac and p-TSA used as catalysts.

The second step of the model study was to evaluate the effect of catalysts on the kinetics of both the target and side reactions. The aim was to determine whether the use of a suitable catalyst could achieve high conversion yield in the target reaction of EP groups with pOH with reduced reaction times in the order of minutes to enable such reactions in a typical twin-screw extrusion process. Ideally, the catalyst would not compromise the negligibility of undesirable side reactions, which has been confirmed in the absence of a catalyst. Figure 5 and the data in Table 3 show that the conversion kinetics of the reaction of SAG8 with CP can be accelerated and EP conversion yields increased with both ZrAcac and p-TSA at typical catalytic concentrations.

**Figure 6:**
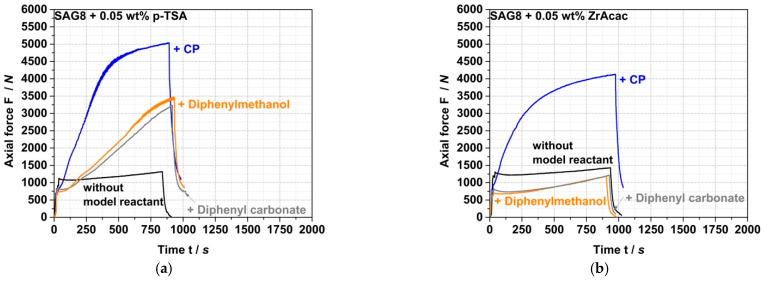
Force vs. time curves measured during compounding of melt mixtures of SAG8 and model reactants of different reactive functionality in the presence of 0.05 wt% of p-TSA (**a**) and 0.05 wt% ZrAcac (**b**), respectively. SAG8 and the model reactants were melt mixed in ratios equivalent to a stoichiometric ratio of the EP-groups in the SAG8 and the monofunctional model reactants.

ZrAcac shows less catalytic activity compared to p-TSA (Figure 5), however, turns out catalytically much more selective in terms of the targeted EP-conversion with pOH-groups (Table 3 and Figure 6). Figure 6 shows the force vs. time curves measured during compounding of melt mixtures of SAG8 and model reactants of different reactive functionality in the presence of 0.05 wt% of p-TSA (a) and 0.05 wt% ZrAcac (b), respectively.

In contrast to the observations with ZrAcac as a catalyst, p-TSA leads to an increase in force with reaction time not only when the SAG8 is reacted with CP (i.e., with pOH groups), but also when it is reacted with diphenylmethanol (i.e., with sec. aOH groups) and even when it is reacted with diphenylcarbonate containing carbonate functionality. Since the increase in force is an indicator of a reaction, and since such an increase in force is not observed when p-TSA is added to pure SAG8 (so that catalysis of EP–EP and EP–nitrile reactions within the SAG8 itself can be essentially excluded), the conclusion is that p-TSA does not selectively catalyze the target coupling reaction of the GMA-containing polymer with pOH, but also the reaction with sec. aOH and carbonate groups. Our interpretation of this finding is that the sulfonic acid group in p-TSA effectively opens the EP ring of the GMA in the SAG8 polymer, thereby non-selectively activating the EP groups for reactions with different kinds of functional groups. This interpretation is confirmed by the increase in EP conversion yields in SAG8 by the addition of p-TSA both in the presence of CP and diphenylmethanol, but rather not in the absence of any low-molecular-weight reactant model compound (Table 3). Moreover, the data displayed in Table 3 show that in the reaction of SAG8 with CP, the p-TSA addition results in an increase in the overall EP conversion, but in no increase in the targeted CP conversion. This observation demonstrates that p-TSA increases the EP conversion by catalyzing the coupling reaction of EP with the sec. aOH groups formed in a prior reaction of EP in SAG8 with the pOH groups in CP (see Figure 1), rather than by catalyzing the targeted coupling reaction with CP itself. Hence, p-TSA does not seem suitable as a catalyst for the eventually targeted melt coupling of pOH-terminated PC with GMA-modified PMMA. 

On the other hand, ZrAcac apparently catalyzes predominantly the target reaction of the EP groups with pOH. In the experiments with SAG8 in combination with either diphenylmethanol or diphenyl carbonate in the presence of ZrAcac (Figure 6b), the forces remained below the values measured for the SAG8 polymer in the absence of any low-molecular-weight reactant model compound—even after rather long reaction times of 15 min. This shows that, in this case, the softening effect related to the presence of unconverted low-molecular-weight model compounds remains predominant during the complete reaction time and proves that ZrAcac does not catalyze, to any significant extent, the undesired side reaction of the EP groups in SAG8 with sec. aOH or carbonate groups. Rather, this catalyst very selectively only accelerates the target reaction of the EP groups with pOH functionalities. In agreement with these observations, ZrAcac as a catalyst leads to a similar in size simultaneous increase in both EP and CP conversion compared to the same experiment without a catalyst (Table 3). 

The overall conclusion of our model investigations with different catalysts is that ZrAcac—although less effective as a catalyst compared to p-TSA in terms of EP activation—is expected to be much more suitable for the intended purpose of catalyzing copolymer formation during the melt compounding of GMA-modified polymers with pOH-terminated PC because of its comparably much higher catalytic selectivity for this specific target reaction. Further experiments were therefore limited to the investigation of ZrAcac as the catalyst. It has to be noted that although the effect of the ZrAcac catalyst on CP conversion is quite limited at the long RT applied in the micro-compounder experiments (increase from 67 to 79% after 15 min; see Table 3), at the much shorter RT, as typical for a twin-screw extrusion process, the effect can be expected to be much more significant.

In the third step of the kinetic model study, we changed the GMA-modified polymer from SAG8 to m-PMMA. The intention was to confirm whether the conclusions previously drawn from the experiments with SAG8 would be valid also for the analogous reaction with the specific type of m-PMMA polymer to be used in the further studies (next section) for the polymer–polymer melt reaction. The experimental data in Table 3 prove that a melt reaction of GMA-modified PMMA with pOH groups in CP occurs as well. However, in the absence of a catalyst, the conversion yield of EP after 15 min is only 56%, which is much lower than in the case of the melt reaction with SAG8 (71%). Obviously, the copolymer-forming target melt reaction is slower in the case of m-PMMA8 than with SAG8. On the other hand, when ZrAcac is added as a catalyst, a similar EP conversion yield of 74% is achieved with m-PMMA8 within 15 min as observed with SAG8 (79%). This means that by using ZrAcac as a catalyst, the differences in the reactivity of EP groups in m-PMMA8 and SAG8 can obviously be compensated. The EP–EP side reaction between two GMA functionalities in m-PMMA8 is negligible both in the absence and presence of the ZrAcac catalyst, as it was in the case of SAG8. Thus, ZrAcac is a promising catalyst candidate for the formation of copolymers by reaction in melt mixtures of GMA-modified PMMA with pOH-functionalized PC. 

The overall conclusion of the kinetic model studies reported in this section is that the reactive modification of BPA-based PC with pOH end groups should, in principle, be a suitable strategy to enable PC copolymer formation via selective coupling reaction with GMA-functionalized polymers in melt mixtures. However, a catalyst might be required to achieve a sufficient reaction speed and thus a high yield of copolymer. ZrAcac is preferred vs. Bronsted acid catalysts such as p-TSA as it exhibits highly selective catalytic activity in the target reaction, thus reducing side reactions that may lead to undesirable crosslinking of the copolymer. In the next section, it will be investigated whether a sufficient reaction speed and thus the desired high PC/PMMA copolymer yield can also be achieved in two-phase polymer–polymer melt mixtures. In this case, the reaction can only take place in the polymer–polymer interphases; thus, the concentration of functional groups viable for reaction is much lower, and the reactive groups may be less available for the copolymer formation reaction due to steric hindrances caused by intra- and intermolecular polymer entanglement.

### 3.2. Reactive Compounding of pOH-Terminated PC with GMA-Modified PMMA in a Discontinuous Process

**Figure 7:**
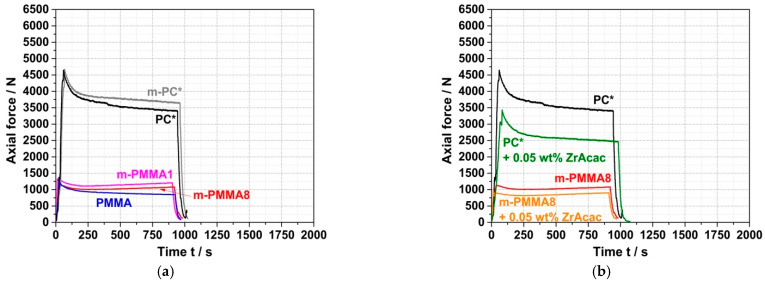
(**a**) Force vs. time curves measured during compounding of the neat polymer blend partners in absence of any catalyst and (**b**) effect of the catalyst (0.05 wt% of ZrAcac) on force vs. time curves measured during compounding of PC* and GMA-modified m-PMMA8.

For reference purposes for the subsequent experiments on the preparation of reactively compounded blends, the force vs. time curves during compounding of the neat blend partners were first recorded in the absence of any catalyst (Figure 7a). As expected, the measured forces and their time dependences are quite similar for the non-reactive commercial PC* and the reactive m-PC* with pOH termination due to their similar molecular weights (Table 1). In both cases, the force decreases slowly with time, which can be attributed to some decrease in molecular weight due to hydrolysis of the PC. According to general expert experience, some molecular weight loss cannot be completely prevented under such melt compounding conditions since trace amounts of water can never be completely eliminated, even in pre-dried PC. When ZrAcac is added to PC, the shape of the force vs. time curve remains essentially unchanged, but the absolute force decreases significantly over the entire timeframe (Figure 7b). We interpret this result to mean that the catalyst softens the PC but does not lead to significantly faster PC degradation than is observed in the absence of the catalyst. The slight decrease in molecular weight during compounding was confirmed by GPC measurements. While the M_w_ of PC* before compounding was 46,000 g/mol (measured using PS calibration; see Table 1), values of 40,000 g/mol and 36,000 g/mol were measured after 15 min of melt compounding at 260 °C in the absence and presence of 0.05 wt% ZrAcac, respectively. M_w_ decreases of this magnitude are typical for commercially available PC under this type of thermal exposure. The effect of ZrAcac on molecular weight degradation can be considered small compared to alternative catalysts that we had previously screened.

Additionally, in the case of PMMA, the reactive (m-PMMA1 and m-PMMA8) and non-reactive (PMMA) polymers behave quite similarly to each other due to their similar molecular weights. However, the measured force values are much lower than those observed when compounding the PC polymers, which is a consequence of the lower melt viscosity of PMMA. The force values are almost not affected by the addition of ZrAcac. For both reactive and non-reactive PMMA, the forces are almost constant over time, regardless of whether ZrAcac is present. Apparently, the molecular weights of PMMA are essentially unaffected by the thermal effects of the compounding process, both in the absence and presence of the ZrAcac catalyst. This interpretation was confirmed by GPC measurements.

**Figure 8:**
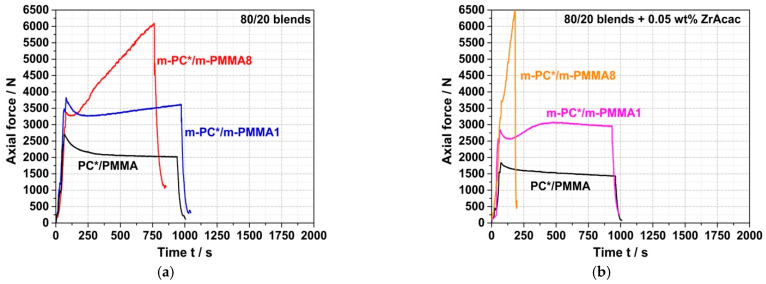
Force vs. time curves measured during compounding at 260 °C of (80/20) PC/PMMA melt mixtures consisting of non-reactive commercial PC* and PMMA polymers and reactive melt mixtures consisting of pOH-terminated m-PC* and GMA-modified PMMA containing 1 wt% (m-PMMA1) or 8 wt% GMA (m-PMMA8) in the absence of any catalyst (**a**) and in presence of 0.05 wt% ZrAcac (**b**).

The force vs. time curves of the conventional PC/PMMA (80/20) blends produced by compounding of PC* with an unmodified PMMA polymer qualitatively look quite similar to those of the neat blend partner investigated before, i.e., force decreases slightly with time, both in the absence of a catalyst and in the presence of ZrAcac (Figure 8). The force levels are in between those observed for the neat blend partners and are lower in the presence of ZrAcac compared to the same composition in the absence of the catalyst. It thus can be concluded that, beyond some PC degradation that is observed independent of the presence of PMMA and/or catalyst, no further (i.e., melt coupling) reaction occurs during the compounding process in this case. 

On the other hand, in the experiments where reactively modified blend partners were used (i.e., m-PC* and m-PMMA1/8), an increase in force was observed during compounding. The velocity (slope) of this force increase depends on the GMA content in the m-PMMA, i.e., it is higher for the blend based on m-PMMA8 than for the blend based on m-PMMA1. In the absence of the catalyst (Figure 8a), no steady state (i.e., either complete reaction or equilibrium) could be reached within 15 min at 260 °C. In contrast, in the presence of 0.05 wt% ZrAcac, the force passes through a maximum after around 8 min in the case of m-PMMA1 as a blend partner (Figure 8b). The slope of the following decrease in force vs. time is similar to the slope observed for the blend of non-reactive commercial polymers. For the same melt compounding experiment with m-PMMA8 in the presence of ZrAcac, a very steep increase in force vs. time is observed (with a slope more than five times higher than for the same experiment in the absence of the catalyst). Due to the technical force limitation of the micro-compounder, the experiment had to be stopped after 3 min to avoid damage to the extruder. At this point, the melt coupling reaction was not yet complete and equilibrium had not yet been reached. However, an approximate doubling of the initial force proves a significant increase in melt viscosity (and thus molecular weight). Considering that side reactions can be essentially excluded based on the results of the model study reported in the previous section, this experimental observation can only be explained by a coupling reaction of pOH-terminated PC and GMA-modified PMMA leading to the formation of a PC/PMMA copolymer. The time required to complete this reaction or reach chemical equilibrium is >>15 min in the absence of a catalyst, but, by using ZrAcac, it can be reduced to the order of <<10 min, which is possible to be realized in a twin-screw extruder. The fact that the melt viscosity increases to higher values at higher GMA content in PMMA can be explained either by the higher PC/PMMA copolymer conversions achieved or by the higher molecular weights of the copolymers realized with PMMA containing a higher amount of GMA units, which allows a higher amount of grafted PC side chains.

**Figure 9:**
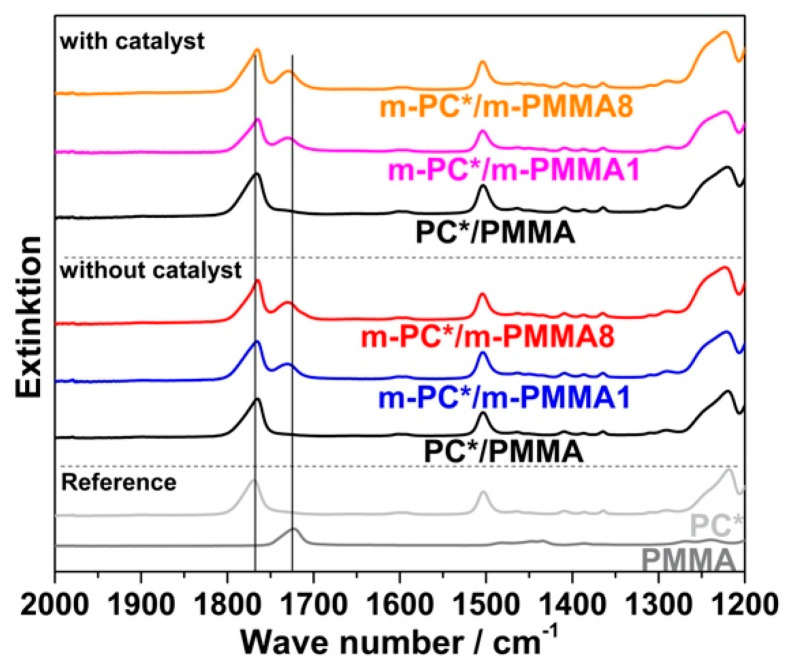
FTIR spectra of the acetone-insoluble parts of PC/PMMA (80/20) compounds produced with non-reactive and reactive blend partners in absence and presence of 0.05 wt% ZrAcac catalyst. The investigated compounds are those produced in the micro-compounder experiments for which force vs. time curves are displayed in Figure 8 (i.e., melt reaction was performed at 260 °C and with a RT in the micro-compounder of 15 min or, in case of the samples with PMMA8, with the maximum RT that was possible based on the force limitations of the machine). The FTIR spectra of the neat PC* and PMMA polymers are included for reference purposes.

The previous conclusion of successful melt coupling of pOH-terminated m-PC* with GMA-modified PMMA m-PMMA1 or m-PMMA8 is confirmed by the FTIR results of the acetone-insoluble parts of the compounds. While only PC is found in the insoluble parts of the compounds produced with non-reactive blend partners, both PC and PMMA are found in the insoluble parts of the compounds prepared with the reactive blend partners—when the compounds are prepared both in the absence and the presence of ZrAcac (Figure 9).

Crosslinking of the GMA-modified m-PMMA due to EP–EP homopolymerization, which could also result in a significant change in its solubility in acetone, can be excluded based on the results of the model study reported in the previous section (observation of negligible EP conversion upon melt compounding of pure m-PMMA8 in both presence and absence of ZrAcac; see Table 3). PMMA crosslinking can be also excluded during the reactive compounding of the reactively modified blend partners based on the observation that the acetone-insoluble parts of the obtained materials were completely soluble in dichloromethane, which is a good solvent for both PC and PMMA, but would not solve any significantly crosslinked polymer. A second alternative interpretation of the spectroscopical finding of PMMA in the acetone-insoluble part of the PC/PMMA copolymer would be copolymer formation via a transesterification reaction of the methyl ester groups of the PMMA with the carbonate linkages of the PC. This interpretation can, however, be excluded as well as it would result in a strong decrease in the PC molecular weight [21], which was actually not observed. 

**Figure 10:**
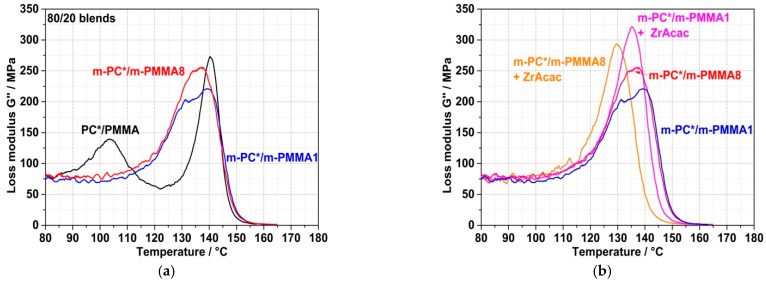
DMA curves of PC/PMMA (80/20) blend compounds. The investigated compounds are those produced in the micro-compounder experiments for which force vs. time curves are displayed in Figure 8. (**a**) Comparison of blends obtained in the absence of a catalyst from non-reactive polymers and from pOH-terminated PC and GMA-modified PMMAs with different GMA contents. (**b**) Effect of use of ZrAcac catalyst (0.05 wt%) during compounding of pOH-terminated PC and GMA-modified PMMAs with different GMA contents.

**Figure 11:**
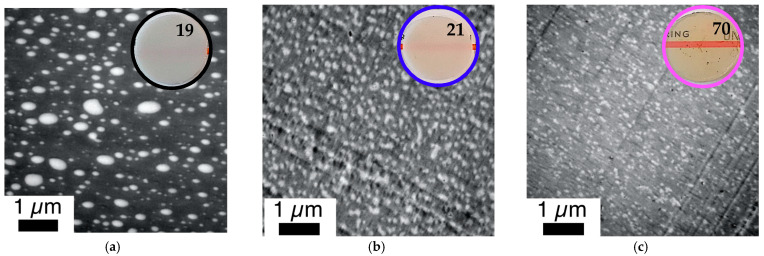
Correlation of phase morphology and transparency of 1 mm thick plaques (numbers represent transmission values determined according to DIN EN ISO 13468-1 on these specimens) prepared by compression molding of various PC/PMMA (80/20) blends. The investigated compounds are those produced in the micro-compounder experiments for which force vs. time curves are displayed in Figure 8. The figure shows a comparison of blends obtained from non-reactive polymers and blends obtained both in absence and presence of ZrAcac, from pOH-terminated PC and GMA-modified PMMA with a GMA content of 1 wt%. (**a**) PC*/PMMA (80/20). (**b**) m-PC*/m-PMMA1 (80/20) (**c**) m-PC*/m-PMMA1 (80/20) + 0.05 wt% ZrAcac.

**Figure 12:**
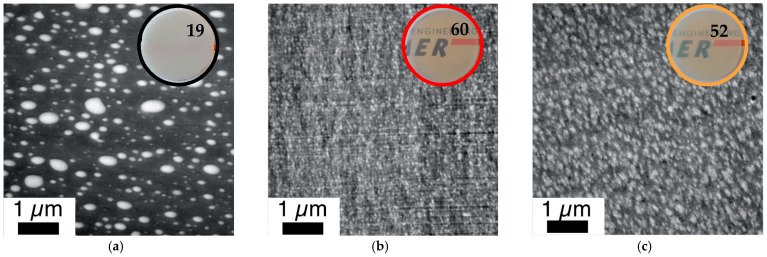
Correlation of phase morphology and transparency of 1 mm thick plaques (numbers represent transmission values determined according to DIN EN ISO 13468-1 on these specimens) prepared by compression molding of various PC/PMMA (80/20) blends. The investigated compounds are those produced in the micro-compounder experiments for which force vs. time curves are displayed in Figure 8. Figure 12 shows a comparison of blends obtained from non-reactive polymers and blends obtained both in absence and presence of ZrAcac, from pOH-terminated PC and GMA-modified PMMA with a GMA content of 8 wt%. (**a**) PC*/PMMA (80/20) (**b**) m-PC*/m-PMMA8 (80/20) (**c**) m-PC*/m-PMMA8 (80/20) + 0.05 wt% ZrAcac.

The spectroscopically proven formation of the PC/PMMA copolymer by the chemical coupling (grafting) reaction on a molecular level obviously causes the compatibilization of the obtained PC/PMMA compounds and thus an improvement in the miscibility of the polymer blend partners. The consequence on a mesoscopic level is a significant change in the phase morphologies of the blends. This can be concluded both from the DMA results shown in Figure 10 and from the TEM micrographs shown in Figure 11 and Figure 12. The compound obtained from non-reactive polymers in the absence of a catalyst shows clearly separated glass transition peaks at 103 °C related to the PMMA and at 140 °C related to the PC. On the other hand, the compounds obtained from pOH-terminated PC and GMA-modified PMMAs, both in the presence and absence of the ZrAcac catalyst, show a single glass transition peak with maxima in the range of approximately 130–140 °C. Such DMA behavior is indicative of a monophase morphology or, at least, of significantly improved phase miscibility/dispersion [34,35]. This is a consequence of a reduction in the interfacial tension at the PC/PMMA boundaries caused by the PC/PMMA copolymer, which is formed in the reactive compounding process. The DMA curve of the compound obtained in the absence of a catalyst from pOH-terminated PC and the m-PMMA1 still shows a qualitative appearance that resembles two strongly overlapping, separate glass transition peaks. One of them has its maximum at 140 °C and seems to be related to a pure PC phase, while the second one has its maximum at around 135 °C, indicative of a miscible PC/PMMA phase. The glass transition peaks of the two compounds obtained in the absence of a catalyst are very broad compared to those of the compounds obtained with the same polymer compositions in the presence of 0.05 wt% of ZrAcac (Figure 10b). The single glass transition with the smallest half-width is observed for the compound obtained in the presence of ZrAcac with the high-GMA-containing m-PMMA8. The peak maximum in this case is at 130 °C and thus in between those of the blend constituents. Our interpretation of this observation is that, in this sample, the maximum and most homogeneous miscibility of PC and PMMA has been achieved. Presumably, the copolymer yield is highest in this material because the kinetics of the copolymer reaction are favored both by the use of the catalyst and by the increase in the concentration of GMA functionalities. 

**Figure 13:**
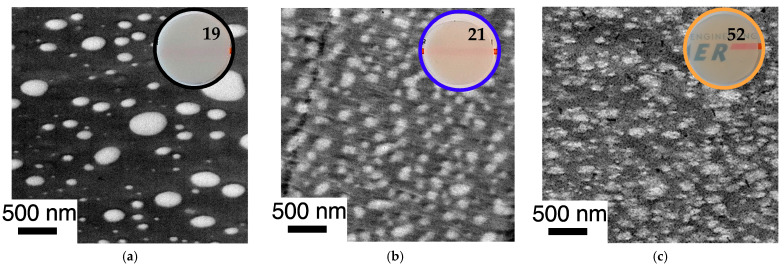
Selected TEM images from Figure 11 and Figure 12 at higher magnification (**a**): PC*/PMMA (80/20); (**b**): m-PC*/m-PMMA1; (**c**): m-PC*/m-PMMA8 (80/20) + 0.05 wt% ZrAcac).

The improvement of the compatibility and hence miscibility of the blend partners due to the in-situ formation of PC/PMMA copolymers, concluded from the results of the DMA experiments, was also confirmed by the TEM investigations (Figure 11 and Figure 12). In agreement with previous literature reports, the reference blend PC/PMMA (80/20) obtained from non-reactive commercial PC and PMMA polymers (Figure 11a and Figure 12a) shows a morphology of PMMA droplets dispersed in a PC matrix. The size of the PMMA droplets exhibits a wide distribution, covering the range from 50 nm to more than 500 nm. The PC/PMMA interphases appear to be very smooth and both polymer phases are well-distinguishable and defined in this case, i.e., the greyscale transition from dark PC to light PMMA in the TEM image is quite discontinuous. This observation suggests that the interphase layers are very thin and the change in chemical composition (i.e., local PC/PMMA ratio) therein is very steep. In comparison, the interphase in the blends obtained by reactive compounding of m-PC* with m-PMMA1/8, regardless of whether ZrAcac was added, all appear to be much rougher and the grey-level transition in the TEM images is much more diffuse. This finding is particularly evident in the TEM image at higher magnification taken for the material prepared with m-PMMA8 in the presence of ZrAcac (Figure 13c). It is less pronounced at lower GMA content in PMMA and in the absence of ZrAcac (Figure 13b). We consider this observation to be indicative of a thicker interphase layer in which the composition of the PC/PMMA ratio changes gradually rather than discontinuously from PMMA in the dispersed domains to PC in the matrix. For the PC/PMMA (80/20) blend obtained in the presence of the ZrAcac catalyst with m-PMMA8, the TEM images show very diffuse greyscale values throughout the material, i.e., also within the PC matrix and the PMMA domains dispersed therein. This behavior seems to indicate that PMMA and PC form an “interpenetrating” phase morphology, where part of the PMMA is an integral part of the PC matrix and vice versa (Figure 13c). This morphology is thus characterized by the occurrence of PC/PMMA ratio gradients not only in the polymer–polymer interphase regions, but in fact throughout the complete blend material. We suggest that the compositional gradients and inhomogeneities are the consequence of a self-organized distribution of the PC/PMMA copolymer, thermodynamically controlled by the characteristic miscibility of the individual copolymer molecules with residual unreacted PC and PMMA polymer. This miscibility, of course, changes continuously with the PC/PMMA ratio in the individual copolymer molecules, i.e., it depends on the number and length of the PC chains grafted onto the PMMA polymer backbone. In addition to these effects, a positive effect on phase dispersion is also evident from the TEM images. The PMMA domain sizes are much more homogeneous and significantly smaller in the blends prepared from m-PC* and m-PMMA1/8 than in the blends of the same ratio prepared from non-reactive polymers. While, for the PC/PMMA (80/20) blends obtained with m-PMMA1, the dispersion is further improved in the presence of the ZrAcac catalyst, the opposite is true for the same blend composition prepared with m-PMMA8 (compare Figure 11 and Figure 12). We believe that the addition of the catalyst to the highly GMA-containing m-PMMA8-based blend, due to higher epoxy conversion, results in crosslinked PC/PMMA copolymers, which are less efficient as phase compatibilizers and thus lead to larger domain sizes. In any case, however, a two-phase morphology with sizes of the dispersed PMMA domains in the order of 100–200 nm is observed for the blends obtained with m-PMMA1 in the absence of ZrAcac and with m-PMMA8 in the presence of the catalyst. For the blends obtained with m-PMMA1 in the presence of ZrAcac and with m-PMMA8 in the absence of the catalyst, the sizes are in the order of <<100 nm.

**Figure 14:**
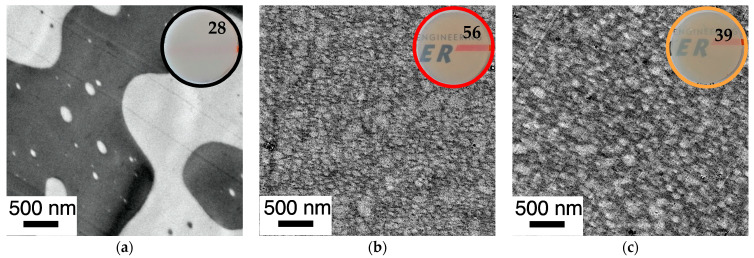
Correlation of phase morphology and transparency of 1 mm thick plaques (numbers represent transmission values determined according to DIN EN ISO 13468-1 on these specimens) prepared by compression molding of various PC/PMMA (50/50) blends. Figure 14 shows a comparison of blends obtained from non-reactive polymers and blends obtained both in absence and presence of ZrAcac, from pOH-terminated PC and GMA-modified PMMA with a GMA content of 8 wt%. The RT in the reactive extrusion production of samples shown in (**b**,**c**) were 12 min and 8 min, respectively. (**a**) PC*/PMMA (50/50) (**b**) m-PC*/m-PMMA8 (50/50) (**c**) m-PC*/m-PMMA8 (50/50) + 0.05 wt% ZrAcac.

The morphologies of the PC/PMMA (50/50) blends show the same trends as described for the PC/PMMA (80/20) compositions (Figure 14). However, the effect of the copolymer on the phase dispersion is even more pronounced in this case. This is because the PC/PMMA (50/50) blend produced from non-reactive polymers shows a co-continuous morphology with much larger PMMA domain sizes (up to in the order of >5 µm) compared to the PC/PMMA (80/20) blends. On the other hand, the PC/PMMA (50/50) blends obtained by the reactive extrusion of m-PC* with GMA-modified PMMA exhibit essentially the same finely dispersed morphologies and similar nanoscale domain sizes as observed for the PC/PMMA (80/20) blends, when compounding is performed both in the absence and presence of the ZrAcac catalyst.

The mesoscopic phase morphology, i.e., the characteristics of the interphase layers, the spatial distribution of PC and PMMA, and the domain sizes of the PMMA phase, as determined by TEM investigations, correlate with the macroscopically observed transparency of the materials (Figure 11, Figure 12, Figure 13 and Figure 14). For both investigated PC/PMMA ratios, the lowest transmission is observed for the blends obtained from the non-reactive polymers, which exhibit the worst PMMA dispersion as well as a very sharp, i.e., discontinuous, PC/PMMA interphase. With transmission values of <30%, these materials are essentially opaque for visible light. The blends with the best PMMA dispersion and thus smallest PMMA domain sizes <<100 nm (i.e., the blends prepared either from m-PC* and m-PMMA1 in the presence of ZrAcac or from m-PC* and m-PMMA8 in the absence of this catalyst) exhibit the highest transparency. Their transmission values range up to 70%, approaching the level measured for the neat blend partners (m-PMMA1: 77%, m-PMMA8: 62% or m-PC**: 81%) (see Table 1). In these cases, the nanoscale domain sizes of the dispersed PMMA phase are roughly one order of magnitude smaller than the visible light wavelengths. This leads to greatly reduced light scattering, regardless of the significant refractive index differences of the blend partners, as expected from the laws of physics and shown in the literature [36,37]. On the other hand, in the cases where the domain sizes of the PMMA phases are in an intermediate range (i.e., in the range of 100–200 nm), the transparency varies greatly, ranging from 21% for the m-PC*/PMMA1 (80/20) blend with 1 wt% GMA in the absence of a catalyst to 52% for the m-PC*/PMMA8 in the presence of ZrAcac. It appears that the high transmission of visible light in PC/PMMA blends having PMMA domain sizes in this range of 100–200 nm is only achieved if an additional structural criterion is met. Based on our results, we suggest that this additional requirement is the presence of continuous PC/PMMA compositional gradients in the interphases of the blend partners [38,39]. This obviously results from and requires the presence of a certain amount of PC/PMMA copolymer. Such continuous gradients in the polymer composition at PC/PMMA interphases result in interphase layers characterized by a steady, i.e., blurred, rather than a discontinuous, i.e., stepwise, change in the local refractive indices. Due to these refractive index gradients, light reflection at the interphases is minimized, thus reducing light scattering and increasing transparency. This mode of action is similar to the operating principle of nanostructured antireflection coatings, which were bionically inspired by the eyes of moths [40,41,42,43]. 

**Figure 15:**
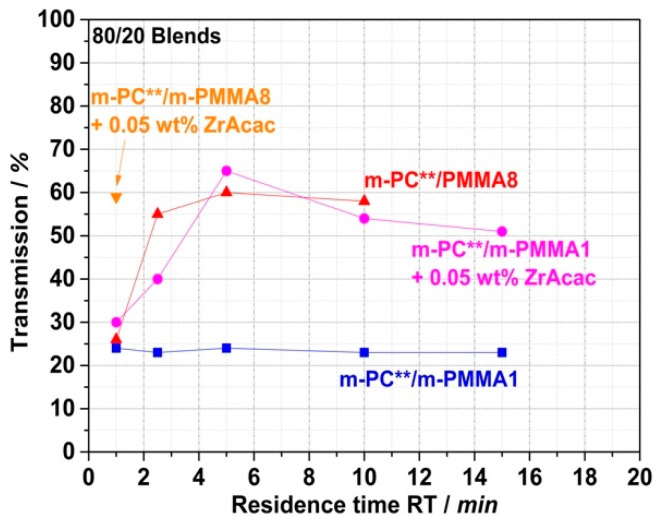
Dependence of light transmission on RT in a discontinuous micro-compounder. Transmission values were measured on 1 mm thick plaques that had been compression-molded from PC/PMMA (80/ 20) blends obtained by reactive melt extrusion at 260 °C of pOH-terminated PC with GMA-modified PMMA in presence and absence of ZrAcac catalyst.

Having successfully shown that translucent or even transparent PC/PMMA blends can, in principle, be achieved at long RT in the order of 15 min, the question remains of whether this goal can also be achieved at much shorter RT in the order of one minute. This is required for typical cost-effective continuous twin-screw compounding processes in industrial manufacturing. It appears to be the case for the compound prepared with the m-PMMA8 in the presence of the ZrAcac catalyst, as it was only melt-compounded for around 2 min before the reaction had to be stopped. However, in the other cases, the results discussed so far do not allow us to draw a reliable conclusion. Therefore, we proceeded with a study of the RT dependence of the transparency obtained in a reactive compounding process in a discontinuous micro-compounder. It should be noted that, for this study, unlike the previous studies, m-PC** was used as the pOH-terminated starting material. m-PC** differs from m-PC*, which was used in the previously reported experiments, in that m-PC** has a slightly lower molecular weight M_w_ and a slightly higher pOH content (Table 1). To ensure comparability with the experiments using continuous twin-screw extruders, which were planned as the next step, we had to change the PC starting material, as m-PC* was not available in sufficient quantities for this purpose. The results presented in Figure 15 show that, in the case of blends based on m-PMMA8, regardless of whether ZrAcac was used, high transmission values >50% can be achieved within a relatively short RT of 2.5 min. In principle, RTs of this size are also technically feasible with conventional twin-screw extruders. However, with an RT of only 1 min, which is more typical for compounding processes with twin-screw extruders, only the composition based on m-PMMA8 obtained in the presence of the catalyst showed this transparency level. To obtain the same result with m-PMMA1 with low GMA content, 5 min RTs are already required, even in the presence of ZrAcac. In the absence of the catalyst, 15 min RTs in this case are still insufficient to obtain any significant transmission increases vs. traditional PC/PMMA blends produced from unmodified polymers. This finding is in line with the results of our previous investigations with the higher-M_w_ m-PC* blend constituent. We thus excluded the experiment with m-PMMA1 in the absence of ZrAcac from the scope of the next step, in which we investigated the transferability of our experimental results to a continuous twin-screw extruder process. 

### 3.3. Reactive Compounding of pOH-Terminated PC with GMA-Modified PMMA in a Continuous Twin-Screw Extruder

In this section, we seek to address the transferability of the previous discontinuous compounding results to a continuous twin-screw extrusion process. This could be then easily scaled up to commercially relevant throughputs using equipment that is broadly established in the compounding industry. For the assessment of the results, it is important to note that the previously used micro-compounder is well-distinguished from the still laboratory-size twin-screw extruder used in the following experiments by the design of the screws (Figure 2). While, in the micro-compounder, the screws are only conveying, the screws of the twin-screw extruder are characterized by the presence of three kneading zones, which are intended to bring in shear energy for improved mixing of the polymer blend partners. This will result in improved dispersion of the PMMA in the PC melt matrix and thus in a mechanically enforced increase in the PC/PMMA interphase area. Since the chemical reaction resulting in PC/PMMA copolymer formation can only occur at this interphase, we can therefore potentially expect increased reaction kinetics and thus higher conversion when compared at the same RT in the case of the twin-screw extrusion process. 

**Figure 16:**
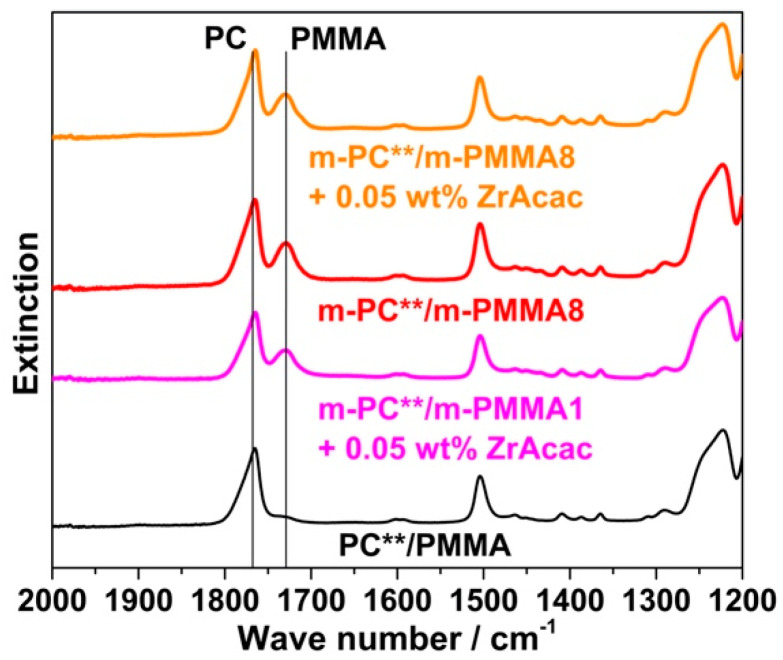
FTIR spectra of the acetone-insoluble parts of PC/PMMA (80/20) compounds produced with non-reactive (black curve) and reactively modified blend partners (colored curves) in absence and presence of 0.05 wt% ZrAcac catalyst. The investigated compounds were produced in a laboratory-scale twin-screw extruder with three mixing zones, with melt temperature set at 260 °C and with an RT of around 1.5 min.

Following the rationale as discussed in the previous section, FTIR spectroscopic results collected on the acetone-insoluble parts of the four different investigated compounds prove that significant amounts of PC/PMMA copolymer are formed upon the melt compounding of reactively modified polymers (Figure 16). On the other hand, the absence of a vibration band at 1725 cm^−1^ related to PMMA in the insoluble part of the compound produced from unmodified PC and PMMA polymers demonstrates that no such PC/PMMA copolymer is formed in this case. 

For the quantification of the amounts of PC/PMMA copolymer formed in the different samples, the same samples were investigated via ^1^H NMR spectroscopy. Table 4 summarizes the integral intensities of the signals related to the eight aromatic protons in the BPA units of the PC (doublet of doublets resonating at chemical shifts in the range of 7.1–7.3 ppm) relative to the intensity of the signal related to the three methyl protons of the methacrylate groups in the PMMA (singlet resonating at a chemical shift of 3.6 ppm). The fractions of PC and PMMA in the acetone-insoluble portions of the reactively compounded blends, which are displayed in the results columns 3 and 4 of Table 4, were calculated from these ^1^H NMR signal intensities. The final column in Table 4 shows the fractions of the 20 wt% PMMA in the blends’ compositions that became part of their acetone-insoluble portions as a consequence of the chemical reaction. These values were calculated assuming that the PC fraction (i.e., 80 wt% of the total blend composition) remains completely insoluble in acetone upon reaction with PMMA. Data show that, by reactive extrusion, we achieved the conversion of up to 73 wt% of the PMMA in the blend into PC/PMMA copolymer, which is the case for the m-PC**/PMMA8 (80/20) blend in the presence of ZrAcac.

**Figure 17:**
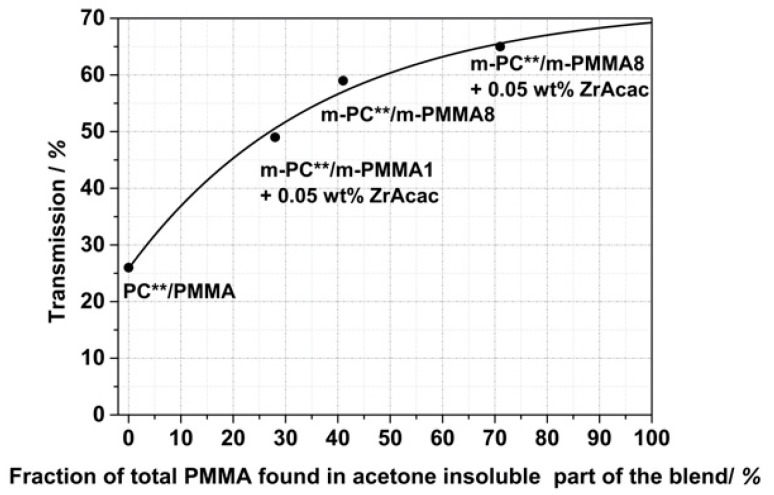
Transmission values measured on 1 mm thick plaques that were injection-molded from PC/PMMA (80/20) blends and correlation with the fraction of the total PMMA in the blend found in the acetone-insoluble part of the compounds by ^1^H NMR spectroscopy. All compounds were obtained in a continuous twin-screw extruder with melt temperature set at 260 °C and with an RT of around 1.5 min.

Figure 17 shows that there is a quantitative correlation between the fraction of the total PMMA in the reactively extruded blend that became an integral part of the acetone-insoluble part as determined by ^1^H NMR spectroscopy, with the level of transparency determined for injection-molded specimens made of the different compounds. As this fraction of PMMA found in the insoluble part is directly related to the content of formed PC/PMMA copolymer, this finding supports our previous hypothesis that transparency in the PC/PMMA blends is a consequence of and requires the formation of the copolymer. By extrapolation of the curve displayed in Figure 17 to 100% conversion of the PMMA, the maximum achievable transmission that can be realized with the reactive compounding technology used in this study can be estimated as in the range of 70%. The limitation here is the transparency of the neat modified PMMA components.

**Figure 18:**
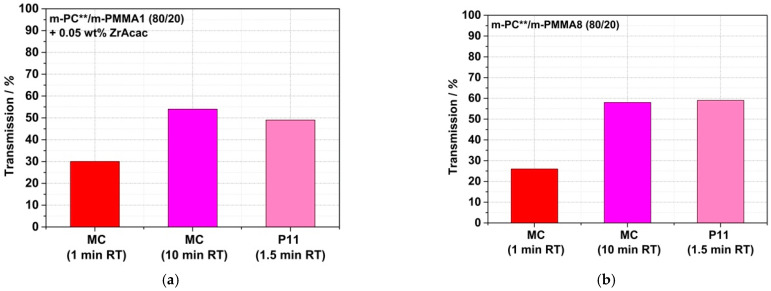

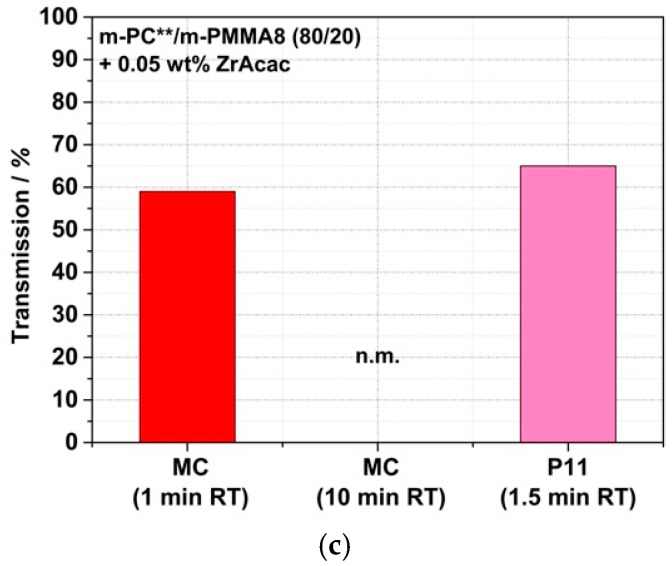
Comparison of light transmission values measured on 1 mm thick plaques made of reactively compounded PC/PMMA (80/20) blends obtained in a discontinuous micro-compounder (MC) at two different RTs and in a continuous twin-screw extruder (P11). Materials were made of pOH-terminated m-PC** and GMA-modified m-PMMA1 in the presence of ZrAcac (**a**) and with m-PMMA8 in the absence (**b**) and presence (**c**) of this catalyst. Test specimens of the materials produced in the micro-compounder were prepared by compression molding of the compounded pellets, while the plaques produced from the materials obtained in the twin-screw extruder were injection-molded.

Figure 18 shows a comparison of the transmission values of reactively compounded PC/PMMA blends that were obtained at different RTs in a discontinuous extrusion process under low shear conditions, with those produced in the continuous twin-screw extruder. As expected, the presence of higher shear in the reactive compounding step allows a reduction in the RT that is required to achieve sufficient copolymer formation to result in blend materials revealing high light transmission. Thus, with the continuous high-shear reactive extrusion process, translucent materials were obtained at RTs as low as 1.5 min. On the other hand, in the case of the blend produced with m-PMMA8 in the presence of the ZrAcac catalyst, for which high transmission had been achieved at an RT of only 1 min already in the discontinuous extrusion, no significant further improvement in the light transmission could be achieved by applying the higher-shear continuous process. 

Having shown that light-transmitting PC/PMMA blends can be prepared by reactive compounding of modified PC and PMMA, the question remains as to how the reactive blending affects the mechanical properties. In previous studies in which transparent PC/PMMA blends had been prepared by the reactive extrusion of unmodified PC and PMMA polymers using a catalytic transesterification approach, the mechanical properties (i.e., elongation at break) of the blends were drastically deteriorated rather than improved. This undesirable observation turned out to be due to the degradation of the PC molecular weight, which is a side effect due to the reaction mechanism [19,21]. The deterioration of the mechanical performance of the blends was so dramatic that the materials had been concluded to be unsuitable for any kind of technical application [21]. 

**Figure 19:**
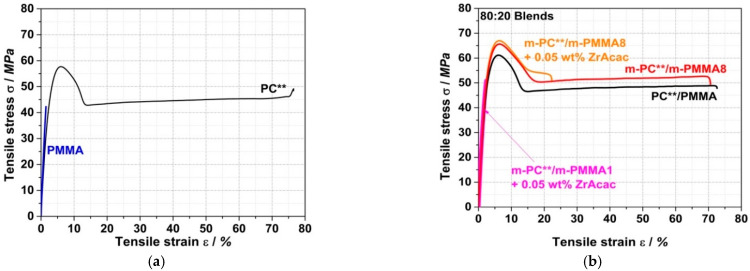
Tensile stress vs. strain curves for (**a**) PC and PMMA polymers and (**b**) traditional and reactively compounded PC/PMMA (80/20) blends. Compounds were all produced in a continuous twin-screw extruder (P11). Test specimens were prepared by injection molding.

Figure 19 shows a comparison of the stress–strain curves from tensile tests on specimens injection-molded from PC/PMMA (80/20) blends prepared by the continuous extrusion process. Table 5 summarizes the characteristic tensile and flexural properties and scratch resistance (as pencil hardness) of the traditional and reactively compounded blends and compares the measured values with those of neat PC and PMMA. 

The most interesting observation in terms of potential technical applications is a synergistic improvement in the scratch resistance of the PC/PMMA blends. Although the blends investigated here predominantly consist of PC, i.e., they contain only 20 wt% PMMA, the measured pencil hardness appears to mostly reflect the performance of hard PMMA (4H) rather than that of the much softer PC (F). Moreover, the reactively compounded blends with a pencil hardness of 3H surprisingly show a further improvement versus the value of 2H measured for the corresponding blend of unmodified PC and PMMA. 

Further synergistic effects are observed in the case of the reactively compounded blends based on m-PMMA8, independently of whether ZrAcac was applied as a catalyst, for the values of ultimate strength σ_fN_ measured for the materials in both tensile and flexural tests. This means that both the ultimate tensile and flexural strengths are higher for these PC/PMMA blends than for each of the individual polymers and also higher than for the conventional blend based on unmodified PC and PMMA. 

No synergistic effects are observed for the moduli in the tensile and flexural tests for the unmodified PC/PMMA blends. Here, within the limits of measurement accuracy, the moduli correspond well to the mass-weighted averages of the moduli of the individual components of the blend in their respective composition ratios. However, a surprising positive effect is evident in the reactively extruded PC/PMMA blends, which exhibit significantly higher tensile and flexural moduli compared to the unmodified PC/PMMA blend. The observed effects can be explained by the improved phase adhesion and thus improved stress transfer between the PC and PMMA phases, which is enabled by the molecular entanglement of the PC/PMMA copolymer in the PC/PMMA interphases of the polymer blend [44,45]. 

In the present case, since the copolymer formation results from melt coupling by nucleophilic addition and not by a transesterification mechanism, the PC molecular weight is not inherently affected in the PC/PMMA copolymer formation reaction. The PC degradation by hydrolysis caused by trace amounts of residual water and driven by the catalytic effect of ZrAcac is also negligible. This explains the higher tensile and flexural strain at break of the PC/PMMA blend obtained by reactive compounding of m-PC** with m-PMMA8 in the absence of ZrAcac, compared to the much lower values reported in previous transesterification studies [21]. 

However, in the two reactively compounded blends prepared in the presence of the catalyst, both the tensile and flexural strains at break are significantly reduced compared to the PC/PMMA blend produced based on unmodified polymers. At this stage of our scientific work, we suspect that the cause of this finding in the case of the blend with m-PMMA8 is the crosslinking of the formed PC/PMMA copolymer, which cannot be avoided at the high number of 18 EP functionalities per polymer molecule and at the high conversion yield of the PMMA of 73% that we achieved (Table 4). On the other hand, we can only speculate at present about the cause of the even lower tensile and flexural strains at break measured for the blend based on m-PMMA1 in the presence of ZrAcac. In this case, the conversion yield of the GMA-modified PMMA is much lower, i.e., 29% (Table 4), and the average number of EP functionalities per polymer molecule in the GMA-modified PMMA is only 2. Thus, crosslinking is much less likely in this case. Speculatively, it could be suggested that the ZrAcac catalyst was not well-dispersed in this particular blend, causing macroscopic inorganic defects in the polymeric test specimens. This may have led to a deterioration in the elongation at break. Further investigations (especially replicate tests) are required to verify our established hypotheses and understand the corresponding surprising observations. 

## 4. Conclusions

Transparent PC/PMMA blends with significantly enhanced tensile/bending performance and scratch resistance could be obtained via the reactive extrusion of terminally pOH-functionalized PC with statistical GMA/MMA copolymers. It was proven by ^1^H NMR and FTIR spectroscopy that the process results in the in-situ formation of PC/PMMA copolymer by the covalent melt coupling of the blend partners. The kinetics of the copolymer formation could be accelerated and thus the copolymer yields enhanced by using ZrAcac as a catalyst. In kinetic model studies, this catalyst turned out to be most suitable, minimizing undesired side reactions such as depolymerization of the polymeric blend partners, homopolymerization of the GMA functionalized PMMA, transesterification and crosslinking via reaction of the EP groups with secondary aliphatic OH groups formed in the course of the target reaction. The transparency of the blends turned out to be strongly dependent on the degree of reactive functionalization of the two constituent polymers, the presence of the catalyst, and the RT of the reactive extrusion process. With a GMA content of the GMA/MMA copolymer of 8 wt%, the RT for achieving a highly light-transmitting PC/PMMA blend could be reduced to around 1 min in the presence of the catalyst. This made it possible to produce a transparent blend with industrially broadly established continuous twin-screw extruder technology. Surprisingly, the transparent blends did not show a single-phase but rather a two-phase morphology of droplet-shaped PMMA domains with a diameter of <<100 nm, which were finely dispersed in the PC matrix. We believe that such a two-phase morphology with phase adhesion enhanced by the copolymer via the polymer entanglement in the polymer–polymer interface of the blend is a prerequisite for the observed improvements in the mechanical properties of the material. In the reactively extruded blends, the interphase layers between the PMMA and PC phases in TEM investigations appeared to be more diffuse than in conventional, uncompatibilized PC/PMMA blends of the same polymer composition. This observation is indicative of a continuous gradient in chemical composition, resulting in a mesoscopic interphase layer by the self-organized distribution of the PC/PMMA copolymer therein. It is postulated that a continuous gradient of refractive indices at the PC/PMMA interphase, in addition to the improvement in the phase dispersion, further supports the reduction in light scattering. Thus, caused by the combination of both contributors, transparency was even achieved in some cases of two-phase PC/PMMA blends with PMMA domain sizes in the range of 100–200 nm. The best balance of high light transmission, scratch resistance, tensile performance, and ductility was achieved in a reactive blend produced in the absence of a catalyst based on pOH-terminated PC and a PMMA containing 8 wt% of GMA units. When a catalyst was added, the PMMA conversion was further increased. However, most likely due to the crosslinking of the formed PC/PMMA block copolymer, this resulted in a deterioration in the tensile strain at break (and thus presumably also ductility), without providing any significant further benefits towards improving the transparency of the material.

## Figures and Tables

**Table 1:** Summary of properties of the used polymers.

Polymer	M_w_/(g/mol) (PS Standard)	M_n_/(g/mol) (PS Standard)	Reactive Group Content	T_g_/°C	Transmission/%
PC *	46,000	27,000	pOH < 0.01 wt%	149	90
PC **	34,000	18,000	pOH < 0.01 wt%	143	90
m-PC *	41,000	23,000	pOH = 0.21 wt%	148	84
m-PC **	34,000	20,000	pOH = 0.27 wt%	143	81
PMMA	61,000	40,000	-	104	97
m-PMMA1	60,000	31,000	EP = 0.3 wt%	109	77
m-PMMA8	60,000	34,000	EP = 2.3 wt%	102	62
SAG8	116,000	60,500	EP = 2.3 wt%	- ***	- ***

* Used in the majority of discontinuous micro-compounder experiments, ** Used in the continuous twin-screw extruder experiments as well as in the RT variation study, which was performed in the discontinuous micro-compounder, *** Not relevant for the current investigation and thus not measured.

**Table 2:** Summary of model compounds used in the reaction kinetic study.

Component	Reactive Group	T_B_/°C	Supplier
4-Cumylphenol (CP)	pOH	335	Merck (Darmstadt, Germany)
1,1-Diphenylethanol	tert. aOH	328	VWR International (Radnor, PA, USA)
Diphenylmethanol	sec. aOH	300	Alfa Aesar (Haverhill, MA, USA)
1-Napthalenemethanol	prim. aOH	300	Merck (Darmstadt, Germany)
Diphenyl carbonate	carbonate	300	Alfa Aesar (Haverhill, MA, USA)

**Table 3:** Summary of the results of the model study of the kinetics of melt reaction of SAG8 and m-PMMA8 polymers with monofunctional model compounds containing different kinds of reactive functional groups. EP and CP conversion yields are reported for different reaction times (RT).

Polymer	Catalyst	Reactant Model Compound	Content of Model Compound/wt%	RT/min	EP Conversion/%	CP Conversion/%
SAG8	-	-	0	15	0	-
SAG8	-	CP	8.0	5	49	45
SAG8	-	CP	8.0	15	71	67
SAG8	-	CP	8.0	30	83	75
SAG8	-	CP	8.0	60	88	82
SAG8	-	1-naphthalenemethanol	6.1	15	5	-
SAG8	-	diphenylmethanol	7.0	15	9	-
SAG8	-	1,1-diphenylethanol	7.5	15	3	-
SAG8	-	Diphenyl carbonate	8.1	15	5	-
SAG8	ZrAcac	-	0	15	0	-
SAG8	ZrAcac	CP	8.0	15	79	79
SAG8	p-TSA	-	0	15	6	-
SAG8	p-TSA	CP	8.0	15	84	68
SAG8	p-TSA	diphenylmethanol	7.0	15	53	-
m-PMMA8	-	-	0	15	0	-
m-PMMA8	ZrAcac	-	0	15	3	-
m-PMMA8	-	CP	10.0	15	56	not measured
m-PMMA8	ZrAcac	CP	10.0	15	74	not measured

**Table 4:** ^1^H NMR results of the acetone-insoluble parts of PC/PMMA (80/20) compounds produced with non-reactive and reactive blend partners in absence and presence of 0.05 wt% ZrAcac catalyst. The investigated compounds were produced in a laboratory-scale twin-screw extruder with three mixing zones, with melt temperature set at 260 °C and with an RT of around 1.5 min.

Sample	^1^H NMR Integral (NMR Integral per Proton)				
	Signal at 3.6 ppm (3H)	Signal at 7.1–7.3 ppm (8H)	wt% PMMA	wt% PC	Fraction of PMMA in Insoluble Part/wt%
PC**/PMMA	0 (0)	8.00 (1.00)	0	100	0
m-PC**/m-PMMA1 + 0.05 wt% ZrAcac	0.54 (0.18)	8.00 (1.00)	6.6	93.4	29
m-PC**/m-PMMA8	0.79 (0.26)	8.00 (1.00)	9.4	90.6	41
m-PC**/m-PMMA8 + 0.05 wt% ZrAcac	1.39 (0.46)	8.00 (1.00)	15.5	84.5	73

**Table 5:** Mechanical properties of traditional and reactively compounded PC/PMMA (80/20) blends as compared to the respective values measured for the PC and PMMA constituent polymers. Compounds were all produced in a continuous twin-screw extruder (P11). Test specimens were prepared by injection molding. E = tensile/bending modulus, σ_fN_ = ultimate tensile/bending strength (* tensile/bending strength at break), ε_B_ = tensile/bending strain at break.

	Bending Test			Tensile Test			Pencil Test
Polymer	E/MPa	σ_fN_/MPa	𝜀_B_/%	E/MPa	σ_fN_/MPa	𝜀_B_/%	Hardness
PC**	2200 ± 20	94.1 ± 0.5	>15	2230 ± 33	58.1 ± 0.1	78 ± 2	F
PMMA	2860 ± 60	65 ± 4 *	2.3 ± 0.2	3190 ± 43	40.9 ± 1.0 *	1.4 ± 0.2	4H
PC**/PMMA	2447 ± 23	98.0 ± 0.4	>15	2586 ± 50	61.1 ± 0.1	76 ± 13	2H
m-PC**/m-PMMA1 + 0.05 wt% ZrAcac	2581 ± 18	64 ± 14 *	2.6 ± 0.7	2827 ± 19	48 ± 3 *	2.2 ± 0.2	3H
m-PC**/m-PMMA8	2519 ± 17	101.4 ± 0.7	>15	2717 ± 28	65.8 ± 0.2	72 ± 9	3H
m-PC**/m-PMMA8 + 0.05 wt% ZrAcac	2559 ± 9	101.5 ± 0.3	>15	2797 ± 15	67.0 ± 0.2	21 ± 3	3H

## Data Availability

The exact data sets of the generated and shown results will be gladly provided upon request.
